# Current Treatment of Comorbid Insomnia and Obstructive Sleep Apnea With CBTI and PAP-Therapy: A Systematic Review

**DOI:** 10.3389/fneur.2018.00804

**Published:** 2018-10-29

**Authors:** Katharina Bahr, Rafael J. A. Cámara, Haralampos Gouveris, Inka Tuin

**Affiliations:** ^1^Department of Otorhinolaryngology, Head and Neck Surgery, University Medical Center Mainz, Mainz, Germany; ^2^Institute of Medical Biostatistics, Epidemiology and Informatics, University Medical Center Mainz, Mainz, Germany; ^3^Department of Psychosomatic Medicine and Psychotherapy, University Medical Center Mainz, Mainz, Germany

**Keywords:** COMISA, sleep apnea, insomnia, PAP-therapy, cognitive behavioral therapy

## Abstract

Insomnia and obstructive sleep apnea (OSA) are often both present in patients with sleep-disordered-breathing (SDB). The coexistence of the two disorders shows an increase in cumulative morbidity and an overall greater illness severity. There is still considerable controversy regarding management decisions in this group of patients. This systematic review focused on more recent evidence regarding treatment of patients presenting with both clinical entities of comorbid insomnia and OSA (COMISA) in terms of their management, especially using combinations of positive airway pressure [PAP, namely aPAP, cPAP, adaptive servo-ventilation (ASV)] and CBTi as well as each one of these two modalities alone. As a conclusion it is necessary to specifically target distinct combinations of both insomnia (initial, middle, late) and OSA (mild, moderate, severe) phenotypes. The present review gives reason to assume that both CBTi and PAP-therapy are necessary. However, it appears that distinct treatment patterns may suit different COMISA phenotypes.

## Introduction

Insomnia and obstructive sleep apnea (OSA) are often both present in patients with sleep-disordered-breathing (SDB) (1–3). An association between insomnia and OSA was first described in 1973 (4). Some studies have shown a high prevalence (39 to 55 %) of insomnia symptoms in patients with OSA in the past (1, 5–7). Insomnia and OSA both share a number of negative consequences, which include increased cardiovascular risk and decreased health-related quality of life (QOL) (8–11). The two disorders combined show an increase in cumulative morbidity and an overall greater illness severity (5). It is believed that OSA could either cause insomnia or exacerbate it (12).

Some studies suggest that the presence of insomnia symptoms may reduce the positive airway pressure (PAP)-compliance in OSA patients (13–16). On the other hand, some studies give reason to believe that insomnia refractory to usual cognitive behavioral therapy (CBTI) may be associated with coexistent SDB. It is also suggested that adequate OSA-therapy leads to improvement of insomnia symptoms (13). Nonetheless, there is still considerable controversy regarding management decisions in this group of patients. The correlation between OSA severity and severity of co-existent insomnia is weak. This fact in combination with a usually indistinguishable clinical presentation of comorbid insomnia and obstructive sleep apnea (COMISA) (17) and insomnia alone raises questions as to whether COMISA is a separate distinct clinical entity at all (18).

To date, the most recent reviews on this topic are from the year 2010 and 2017. In their systematic review from the year 2010, Luyster et al. suggested that the combination of both CBTI and OSA treatment resulted in greater improvements in insomnia than did either CBTI or OSA treatment alone (13). Sweetman et al. reviewed research focusing on prevalence, characteristics and theoretical mechanistic relationships in COMISA patients (17). They emphasize on different insomnia entities, stating that insomnia and its symptoms in COMISA could either be secondary to OSA or an independent entity. Thus, depending on its entity, COMISA needs to be treated differently. However, the differentiation between independent and secondary COMISA is difficult.

We extended the information of these two studies by reviewing literature from 2010 to 2017 focusing on the following aspects:

Our primary research question was to review observational or interventional studies about the association between PAP-therapy and CBTI as exposures and insomnia as the outcome in COMISA patients. A secondary aim was reviewing observational or interventional studies about insomnia as exposure and adherence to PAP-therapy as the outcome. A tertiary aim was reviewing the comparators of PAP-therapy and CBTI on the one hand and adherence to PAP-therapy on the other, in order to make recommendations for future research.

## Methods

### Literature search

Eligible studies included populations with OSA and insomnia. Acceptable definitions of OSA were an apnea-hypopnea-index (AHI) ≥5 events/hour or a respiratory-distress-index (RDI) of ≥5 events/hour. Acceptable definitions of insomnia were an insomnia severity index (ISI) ≥ 14 points, a Regensburg-Insomnia-Score (RIS) ≥ 14 points or a Nordic sleep questionnaire ≥ 4 points on one of the insomnia questions. Eligible studies included interventions of PAP and/or CBTI starting at study beginning or prior. Pharmacological and surgical interventions were out of the scope of this review. Comparators were presence vs. absence or different levels of OSA or insomnia. Although the focus of this review was the direct association between OSA and insomnia, outcomes such as adherence to the intervention were also eligible, as long as they referred to OSA or insomnia. Outcomes related to other chronic conditions, such as dementia or multiple sclerosis, were an exclusion criterion. Eligible study designs were interventional studies as well as cohort studies, case control studies and case series.

The Medline terms for the identification of literature were [(insomnia asv) OR (insomnia pap) OR (insomnia apap) OR (insomnia cpap) OR (CBTI OSA) OR (cognitive behavioral therapy OSA insomnia) OR (CBTI sleep apnea insomnia) OR (cognitive behavioral therapy sleep apnea insomnia)] AND (“2010/01/01”[PDat]: “2017/10/15”[PDat]). During the selection process, the first and third author classified the search results according to a protocol established by the second author. They performed a title/abstract screening followed by a full text screening and met after each round to solve disagreement regarding eligibility and main reason for exclusion in consensus. The study selection protocol followed the PICOS algorithm (population, interventions, comparators, outcomes, and study designs). The language was restricted to English or German articles. The first and third author screened the selected articles for further eligible studies.

### Extraction and presentation of data

Following a PICOS-based protocol, the first and second author extracted data about methods and results of the original articles and presented it in two tables. In case of multiple exposures/interventions and outcomes related to OSA or insomnia as well as multiple analyses of one association, they presented the data most related to the primary objective of this systematic review, which was the direct association between OSA and insomnia. Second, they favored exposures/interventions clearly preceding the outcome. Third, they preferred results from thorough analyses with straightforward association measures supported by confidence intervals and *p*-values. Based on quantitative information, the authors made an effort to provide their own interpretation of the results rather than blindly repeating statements from the discussions of the original articles. They solved disagreements between each other in consensus and summarized interpretational deviations from the conclusions of the original articles in the text. They summarized characteristics of the original samples with standard measures. Due to a majority of observational original studies, they followed the MOOSE checklist [(19); Supplementary Material] and completed it with the PRISMA checklist (20), if necessary.

## Results

### Methodological issues

**Figure 1:**
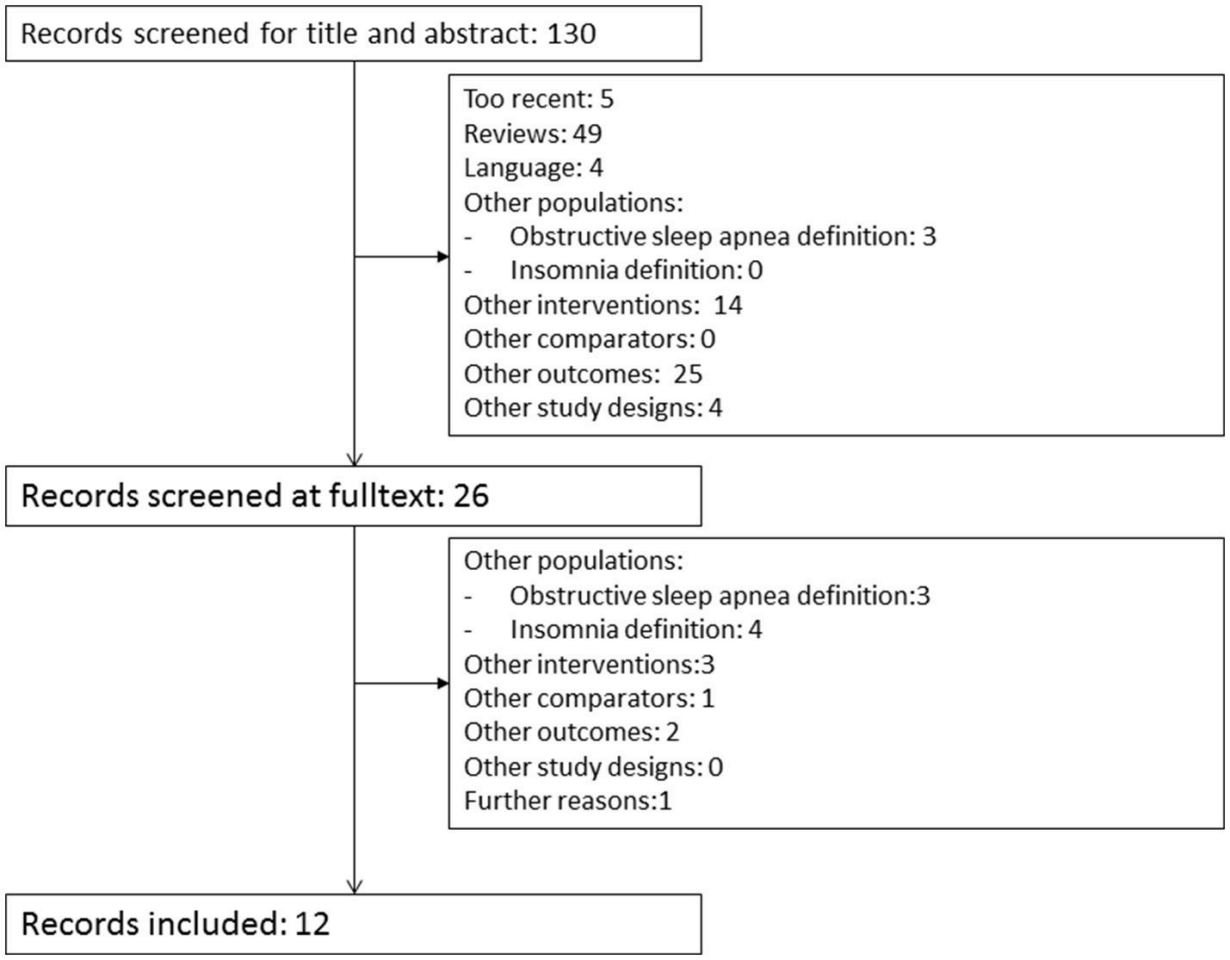
Literature selection process.

Of 130 records identified in Medline, 12 studies were eligible (Figure 1). Additional handsearch revealed another study eligible to our research question (21). Of these 13 studies, six addressed our primary research question (21–26) and the other seven analyzed the association between insomnia as exposure and adherence to PAP as outcome (12, 27–32). To define OSA, seven studies used the AHI with three different cutoffs, three used the RDI with two cutoffs, one study used both RDI and AHI, and two relied on the international classification of sleep disorders. In order to define insomnia, seven studies used the Insomnia Severity Score (ISS) with three different definitions. Two studies used the Basic Nordic sleep questionnaire, two relied on the international classification of sleep disorders and one study used the RIS. Ten studies had PAP-therapy as the only intervention, two studies investigated CBTI alone, and one study examined both therapies. Four studies differentiated between initial, middle and late insomnia (Table 1). Three of the six studies that addressed our primary research question and two of the seven studies that analyzed the association between insomnia and adherence to PAP used more eligibility criteria than OSA and insomnia to define their populations. In the former group, one study included only military veterans with a mini mental state ≥24 points and without serious physical or mental health issues (24), one included only patients with psychophysiological risk factors of insomnia such as poor sleep hygiene (25), and one excluded other sleep disorders such as narcolepsy (26). In the latter group, two included only military veterans without previous OSA-surgery or oxygen supplementation (27, 30). Two studies included participants from the icelandic sleep study for different objectives (28, 32). Their data probably overlapped.

**Table 1:** Methods of the included studies on insomnia, obstructive sleep apnea, and positive airway pressure therapy/cognitive behavioral Therapy.

**References**	**Population (eligibility criteria)**				**Exposure/intervene-tion**	**Comparison/control**	**Outcome**	**Study**		
	**Obstructive sleep apnea**	**Insomnia**	**Positive airway pressure (PAP)**	**Cognitive behavioral therapy**				**Type**	**Design**	**Measurement time points**
Nguyen and Chaskalovic (12)	Respiratory distress index ≥10 events/hour	Insomnia severity index ≥ 14	From study beginning; auto-adjusting	No	Insomnia at baseline	No insomnia at baseline	Continuous therapy adherence in minutes/night in the last 4 weeks	Observation	Cohort study	Baseline, 1 month, 6 months
Wickwire et al. (27)	Diagnosis of obstructive sleep apnea	Insomnia severity subscale score (first 3 questions) ≥ 4	From study beginning; continuous	No	Initial, middle and late insomnia	No initial, middle, late insomnia	≥ 4 h/night in 70% of the nights in the last 4 weeks vs. less therapy adherence	Observation	Cohort study	Baseline, 28 up to 365 days
Björnsdottir et al. (28)	Apnea-hypopnea-index ≥15 events/hour	Basic nordic sleep questionnaire (1 item ≥ 4)	From study beginning; auto-adjusting, continuous, bilevel and adaptive servoventilation	No	Initial, middle and late insomnia	No initial, middle and late insomnia	≥ 4 h/night in 70% of the nights in the last 4 weeks vs. less therapy adherence	Observation	Cohort study	Baseline, 2 years
Nguyen et al. (22)	Apnea-hypopnea-index ≥10 events/hour	Insomnia severity index ≥ 15	From study beginning; auto-adjusting	No	≥ 240 min auto-adjusting positive airway pressure/night	Less hours or discontinuation	Insomnia improvement from baseline to follow-up ≥ 9 points	Observation	Cohort study	Baseline, 24 month
Pieh et al. (29)	International classification of sleep disorders-2	Regensburg Insomnia Scale	From study beginning; continuous	No	Continuous change for one insomnia point at baseline		Continuous therapy adherence in minutes/night (period not specified)	Observation	Cohort study	Baseline, 6 months
Wallace et al. (30)	International classification of sleep disorders-2	Insomnia severity index ≥ 15	512 days ± 484 prior to baseline; continuous	No	Continuous change for one insomnia standard deviation at baseline		Continuous therapy adherence in minutes/night over the entire period	Observation	Cross-sectional study	Baseline
Glidewell et al. (23)	Apnea-hypopnea-index >5 events/hour or respiratory distress index >15 events/hour	Insomnia severity subscale score (first 3 questions) ≥ 4	From study beginning (unspecified devices)	No	Exploration of different predictors, of which minutes auto-adjusting positive airway pressure/night and the respiratory distress index at baseline were the most related to our research objective		Improve-ment vs. persistence of insomnia (<4 vs. ≥4 at follow-up)	Observation	Cohort study	Baseline, 43 ± 7.1 days
Wohlgemuth et al. (31)	Diagnosis of obstructive sleep apnea	Insomnia severity index	Up to 5 years prior to study beginning	No	Exploration of different predictors, of which insomnia and the respiratory distress index at baseline were the most related to our research objective		Non-adherers, attempters and adherers according to a cluster analysis	Observation	Cross-sectional study	Baseline
Eysteinsdottir et al. (32)	Apnea-hypopnea-index >15 events/hour	Basic Nordic sleep questionnaire (1 item ≥ 4)	From study beginning; auto-adjusting and continuous	No	Initial, middle, late insomnia at baseline	No initial, middle, late insomnia at baseline	Quitting the therapy ≤ 1 year vs. quitting later (non-quitters excluded)	Observation	Cohort study	Baseline, 6,7 ± 1.2 years
Fung et al. (24)	Apnea-hypopnea-index <15 events/hour	International classification of sleep disorders-2	No	From study beginning for 6 weeks	Mild obstructive sleep apnea (apnea-hypopnea-index ≥ 5 events /h)	No obstructive sleep apnea (apnea-hypopnea-index <5 events /h)	Sleep improvement	Intervention	Randomized Cognitive behavior-ral therapy or sleep education	Baseline, 6 weeks, 6 months, 12 months
Krakow et al. (25)	Apnea-hypopnea-index >5 events/hour or respiratory distress index >15/h	Insomnia severity index ≥ 15	More than 6.9 months prior to study beginning auto-adjusting and adaptive servoventilation	unknown	Full users of therapy (≥ 20 h/week, probably last 4 weeks)	Partial users of therapy	Continuous change of subscale scores for initial, middle and late insomnia between the baseline and the previous visit	Observation	Case series	Baseline
Ong et al. (26)	Apnea-hypopnea-index ≥ 5 events/hour	International classification of sleep disorders-2	From study beginning	From study beginning	Positive airway pressure therapy or/and cognitive behavioral therapy	None	Insomnia severity index, total wake time in minutes	Observation	Cohort study	Baseline, 90 days
Sweetman et al. (21)	Apnea-hypopnea-index ≥ 11 events/hour or respiratory distress index ≥15/h	Insomnia according to the diagnostic and statistical manual of mental disorders IV and V	No	From study beginning	Obstructive sleep apnea at baseline	No obstructive sleep apnea at baseline	Continuous insomnia change from baseline to 3 months	Observation	Cohort study	Baseline, post-treatment, 3 months

### Outcomes

Of the six studies addressing our primary research question, four indeed analyzed the effect of PAP, i.e., OSA-therapy, on insomnia (22, 23, 25, 26), while two studies analyzed the direct effect of OSA on insomnia comparing two groups without PAP, and with equal CBTI for both groups (21, 24). Of the four studies analyzing the effect of PAP on insomnia, three measured the exposure in minutes per night and one used no control. Of the former three studies, two used a cut-off, namely 240 min PAP-use per night and 171 min per night (22, 25), and the third looked at the exposure continuously (23). The second only reported results for participants above the cut-off stating that those below were not statistically significant (26). One defined the outcome as improvement of the ISI of 9 points (22), one defined it as follow-up subscale score of <4 of the ISI (23), and one reported no definition. The last differentiated between initial, middle, and late insomnia (25). The first found that adherence to OSA therapy increased the chance of successful insomnia therapy by 1.1 times (22), the second found that patients with persistent insomnia have 72 min less PAP-use per night (23), and the study omitting the results for the control found improvements of 0.7 standard deviations in initial, 0.9 in middle, and 0.6 in late insomnia (25). The study without control for the exposure found that PAP and/or CBTI decreased insomnia by 0.55 standard deviations and the total wake time by 41 min on the average (26). The findings were statistically significant in the second and the third of these four studies. The studies analyzing the direct effect of OSA on insomnia were the only interventional studies that fitted our eligibility criteria. The first one found that insomnia decreased more in participants randomized to sleep education than those randomized to CBTI (24). Besides, it found that the difference between sleep education and CBTI was 21 min higher in participants with mild OSA than in those without. This interaction was not statistically significant (Table 2) (24). The second study dealing with CBTI found that CBTI decreases insomnia by 2 points less and increases sleep efficiency by 1.8% more in patients with OSA compared to those without (21). Generally, the robust clinical improvements with CBTI were comparable between those with insomnia alone and the COMISA patients.

**Table 2:** Results of the included studies on insomnia, obstructive sleep apnea, and positive airway pressure therapy/cognitive behavioral therapy.

**References**	**Population (characteristics of the sample)**					**Association between exposure/intervention and outcome**				
	**Sample size**	**% of women**	**Age in years (mean ±standard deviation)**	**Body mass index in kg/m^2^ (mean ±standard deviation)**	**Apnea-Hypopnea-Index/Respiratory Distress Index in events/hour (mean ±standard deviation)**	**Association measure**	Strength of the association^**^	**Interpretation**	**Confidence Interval**	***p*-value**
Nguyen and Chaskalovic (12)	148	18.2	54.8 ± 11.8	29.1 ± 6.3	39.0 ± 21.3	Unadjusted mean difference at 6 months	24	Insomnia decreases the adherence by 24 min/night	Missing	Not significant (*p*-value missing)
Wickwire et al. (27)	232	43.5	53.6 ± 12.4	43.4 ± 7.7	41.8 ± 27.7	Odds ratios of adherence adjusted for age and gender	Initial: 0.95 Middle: 0.81 Late: 1.07	Initial insomnia decreases the chances of adherence by 1.05, middle insomnia by 1.23; Late insomnia increases the chances by 1.07	Missing	Initial: 0.55 Middle: 0.02 Late: 0.53
Bjornsdottir et al. (28)	705	19.4	54.9 ± 10.2	33.7 ± 5.6	45.5 ± 20.5	Odds ratios of adherence adjusted for sex, age, body mass index, and obstructive sleep apnea severity	Unadjusted: −0.56 for initial for middle 0.53 for late Adjusted: 0.59 for initial 0.98 for middle 0.55 for late insomnia	Initial and late insomnia almost halves the chances of adherence, while middle insomnia has no effect	Adjusted 95 % 0.38–0.91 for initial 0.70–1.37 for middle 0.39–0.79 for late insomnia	Initial: 0.01 Middle:0.89 Late: <0.001
Pieh et al. (29)	73	32.9	55.1 ± 11.5	30.8 ± 5.0	39.2 ± 26.7	Linear regression coefficient adjusted for statistically significant univariate correlations^*^; Pearson-Correlation between exposure and outcome at 6 months	0.347 h per insomnia point on a standard deviation of 7.5 points; 0.12	Adherence to therapy diminishes by 156 min/night for one standard deviation of insomnia, which explains 12% of its variance	Missing	0.007
Nguyen et al. (22)	80	12.5	54.9 ± 10.6	30.5 ± 6.0	45.0 ±24.6	Odds ratio of response to therapy on adherence to therapy adjusted for age and body mass index, Epworth sleepiness score and respiratory distress index	1.124	Adherence to therapy increased the chance of insomnia responding to therapy by 1.124 times	0.986–1.280	Missing; non-significant according to the 95 % confidence interval
Wallace et al. (30)	248	6.0	59.0 ± 11.0	33.0 ± 5.0	40.0 ± 30.0	Standardized linear regression coefficient in daily hours of positive airway pressure use adjusted for race, OSA severity, CPAP adherence download variables and sleep related questionnaire responses	−0.28	Adherence to therapy diminishes by 17 min/night for one standard deviation of insomnia	Missing	<0,001
Glidewell et al. (23)	68	32.4	47.5 ± 12.4	32.2 ± 7.3	34.7 ± 32.2	Differences of means (standard deviations) between both groups adjusted for total number of medications, medical and psychiatric diagnoses	Average PAP use: 71.6 Respiratory distress index: 17.8 (40)	Patients with persistent symptoms have 72 min less PAP-use per night and 0.4 standard deviations less respiratory distress at baseline	Missing	PAP-use: 0.02 Respiratory distress index: 0.01
Wohlgemuth et al. (31)	207	6.3	58.4 ± 11.9	32.4 ± 5.0	40.0 ± 29.4	Odds ratio for attempters and adherers vs. non-adherers adjusted for age and years	Insomnia: 0.956, 0.870 Apnea-hypopnea -0.55-index: 0.977, 1.003	Insomnia decreases the chance of being an attempter by 1.046 and the chance of being an adherer by 1.149	Missing	Insomnia: 0.248, 0.004 Apnea-hypopnea-index: 0.005, 0.722
Eysteinsdottir et al. (32)	796	19.1	54.4 ± 10.6	33.5 ± 5.7	44.9 ± 20.,7	Odds ratio for quitting therapy early adjusted for OSA severity, daytime sleepiness, age and gender	Initial insomnia: 2.03 Middle insomnia: 0.99 Late insomnia: 1.75	While late and middle insomnia has no influence on being an early-quitter, initial insomnia doubles the chances.	Initial: 1.17–3.52 Middle: 0.63–1.56 Late: 1.09–2.82	Missing; initial and late insomnia significant according to the 95% confidence
Fung et al. (24)	134	3.0	72.2 ± 7.7	Missing	9.4 ± 5.3	Unadjusted mean difference of total wake time minutes in post-treatment effect between mild and no obstructive sleep apnea	21.3	The advantage of sleep education over cognitive behavioral therapy increases by 21 min in mild obstructive sleep apnea	−54.3 to +96.8	0.58
Krakow et al. (25)	302	54.4	53.4 ± 14.2	31.6 ± 8.0	32.0 ± 28.2	Cohen's ds of insomnia improvement in full PAP-users (partial users missing because not significant)	Initial: 0.70 Middle: 0.87 Late: 0.65	In full PAP-users initial insomnia improved by 0.7, in middle by 0.87 and in late by 0.65 standard deviations	Missing	<0,01 for ANOVA
Ong et al. (26)	32	61.8	54.1 ± 13.3	37.6 ± 10.9	35.3 ± 31.6	Unadjusted Cohen's ds between baseline and follow-up for insomnia; Mean difference for total wake time	Insomnia severity index: −0.55 Total wake time: −41	The therapy decreases insomnia by 0.55 standard deviations and total wake time by 41 minutes	Missing	Insomnia: 0.002 Total wake time: 0.003
Sweetman et al. (21)	455	66.9	51.7 ± 15.7	26.3 ± 4.9	14.3 ± 8.0	Unadjusted differences of means between groups for insomnia and sleep efficiency	Insomnia severity index: 2.4 Sleep efficiency: 1.8%	Therapy decreases insomnia by 2 points less and increases sleep efficiency by 1.8% more in patients with obstructive sleep apnea than in patients without	For the within group estimators: ± 1.8 (exposure), ± 1.1 (control) for insomnia; ± 3.7 (exposure), ±2.4 (control) for sleep efficiency	0.011 unadjusted for insomnia (adjusted not significant), 0.156 unadjusted for sleep efficiency

* Age, sex, educational level, body mass index (BMI), intake of hypnotic medication.

** *Whenever applicable, exposure minus or divided by control*.

In the six studies addressing our primary research question, the proportion of women ranged between 3.0 and 66.9%, the mean age ranged between 47.5 and 72.2 years, and the AHI/RDI ranged between 9.4 and 45.0 events per hour. In the seven studies addressing the effects of insomnia on adherence to PAP, the proportion of women ranged between 6.0 and 43.5%, the mean age ranged between 53.6 and 59.0 years, and the AHI/RDI ranged between 39.0 and 45.5 events per hour. Three of them differentiated between initial, middle and late insomnia (27, 28, 32). Four studies compared presence of insomnia according to criteria summarized above vs. absence of insomnia (12, 27, 28, 32). The other three looked at the exposure as a continuous predictor (29–31). With respect to the outcome, five of seven studies measured PAP-adherence in minutes per night (12, 14, 28–30), one defined non-adherers, attempters, and adherers by cluster analysis (31), and one compared participants quitting the therapy before 1 year after initiation with those quitting later and excluded the continuers (32). Of the first five, three looked at insomnia continuously, where one found that insomnia decreases the adherence by 24 min per night (12), one found that one standard deviation of insomnia diminishes it by 156 min per night (29), and the last one found that the standard deviation decreases it by 17 min per night (30). Two divided insomnia into three phenotypes, where one found that initial insomnia decreases the chances of adherence by 1.05, middle insomnia by 1.23 and that late insomnia increases the chances by 1.07 (27), and one found that initial and late insomnia almost halves the chances of adherence, while middle insomnia has no effect (28). The study that defined adherence by cluster analysis found that insomnia has no effect on being an attempter and decreases the chance of being an adherer by 1.1. The last study found that late and middle insomnia has no influence on being an early-quitter, while initial insomnia doubles the chances (31) (Table 2).

## Discussion

Two of four studies reporting controlled results are too few to draw well-supported conclusions on the influence of the OSA-therapy on insomnia (22, 23), and two clinical trials reporting an interaction between OSA and the effect of insomnia-therapy on insomnia is little evidence for or against the influence of OSA itself on insomnia (21, 24). While the first of the two controlled observational studies found a very small effect of OSA-therapy on insomnia (OR 1.1, 95%CI: 1.0 to 1.2) (22), the second found that increasing PAP-use by 72 min per night may favor the improvement of insomnia (*p* = 0.02) (23). It remains uncertain, however, how likely this additional PAP-use might improve insomnia. One of the clinical trials found that the presence of OSA increases insomnia by 21 min per night, more precisely, it detected the superiority of sleep education over CBTI that increases so much if the patient has OSA (24). Reporting this interaction, the direct effect of OSA on insomnia was omitted. The 21 min are neither clinically relevant nor statistically significant (*P* = 0.58). Altogether, the small number of studies plus the inconsistency and incompleteness of the results provide little evidence about a potential association between OSA-therapy and insomnia.

With seven controlled observational studies, the effect of insomnia on PAP-adherence is better investigated than the effect of OSA-therapy on insomnia. However, four studies with clinically relevant associations [156 min decrease of adherence per night, 1.23 lower, and halved chances of being adherent (27–29, 32)] against three studies with negligible associations (24 and 17 min decrease per night, 1.1 lower chances) (12, 30, 31) favor neither one conclusion nor the other. The strongest associations (halved chances of being adherent) favor a decrease of PAP-adherence caused by insomnia. As all studies distinguishing between initial, middle and late insomnia found very different associations regarding these three entities, this distinction seems important (25, 27, 28, 32). One study found clinical relevance in middle insomnia only, which decreased adherence (OR 1.2) (14), one found high clinical relevance in initial and late insomnia, which decreased adherence (OR 2) (28), and one found high clinical relevance again for initial insomnia, which decreased adherence (OR 2) (32). Taken together, the association with late insomnia seems the strongest. However, the evidence is again too small for conclusions.

At study level, heterogeneous populations, distinct definitions and measurements of exposures/interventions, outcomes and controls, and different analyses might be some reasons for the heterogeneity of the findings. The thirteen studies used seven different definitions of OSA and eight different definitions of insomnia. However, most of these definitions match well, and most studies used PAP-therapy. The most striking heterogeneity in terms of populations is that three studies included only military veterans with a percentage of women ranging between three and six vs. 12 and 62 in the other studies (24, 30, 31). Two of these three populations had a similar age distribution as the other studies, and one was clearly elder (72 years with a standard deviation of eight) (24). The most probable reason for that was the fact that this study sampled insomnia patients and screened them for OSA, while the other studies sampled OSA-patients and screened them for insomnia. In conclusion, the populations were heterogeneous but still comparable. The fact that three studies measuring adherence to PAP-therapy as exposure and the seven measuring adherence to PAP-therapy as outcome used seven very different definitions of adherence to PAP-therapy compromised the comparability more than the different populations. The remaining three used CBTI and a mix of CBTI and OSA.

At review level, two main limitations may contribute to the small number of studies and to the incompleteness of the results. First, we relied on Medline as the only search engine; second, we had no contact with the authors when information was missing or our and their interpretation of the findings differed. However, our structured approach included more than 100 working hours per person in a thorough section of literature and data extraction following the PICOS algorithm. A publication period ranging from the last original article of the updated review by Luyster et al. up to 6 months before identifying the literature reduced the publication bias, because search engines rapidly process publications that are easy to find, but need several months to process publications with lower impact factor. The entire search was double-checked and two investigators discussed and interpreted all findings together without merely relying on statements from the discussions of the original articles. While they did so, they found that the cited research confirmed the completeness of the identified literature. A further strength at review level at the expense of the number of eligible studies was a satisfactory definition of the population as inclusion criterion. It resulted in the exclusion of studies confirming insomnia by superficial questions such as “do you have insomnia.” Overall, the ratio between efforts to improve quality on the one hand and availability information on the other was considerable.

The review by Luyster that we updated found two studies with OSA-therapy as exposure and insomnia as outcome, as well as one study with insomnia therapy as intervention and adherence to PAP-therapy as the outcome (13). As it used no defined publication period as inclusion criterion, the period was probably longer than ours. Nevertheless, the previous review found fewer studies fitting our other criteria than we did, which also points out the comprehensiveness of the literature that we identified. Of the first two studies, one was a pilot study attaining a non-clinical level of insomnia in eight of 17 patients with CBTI alone. After adding OSA-therapy, further seven of the remaining nine patients attained a non-clinical level (33). In other words, this before after study, just as two of our four studies, had no control. The other found that the combination of OSA-surgery and CBTI was the best option for COMISA (34). This surgical study was out of the scope of our PAP-review. The third study found regular PAP-use in 26 of 39 patients assigned to a daytime sleep medical procedure compared to 14 of 60 historical controls (35). This very impressive finding corresponds to an OR of six and makes our ORs of two for the effect of initial and late insomnia on adherence and for the effect of initial insomnia on non-adherence looking small. It is surprising, because the treatment of the disease in the best case annuls 100% of the impact of the disease itself. It would have been interesting to know how the daytime sleep medical procedure influenced initial, middle, and late insomnia itself. In other words, we found thin evidence but the evidence of the studies we updated was even thinner. An effect of insomnia on adherence to PAP-therapy is probable, however.

The more recent review by Sweetman et al. found in total 8 studies dealing with either CBTI alone or with combined treatments in COMISA patients including drug studies, case studies and studies dating back up to the year 2001 (17). One study in the review by Sweetman et al matched our well-defined eligibility criteria, namely the study by Lack et al. presented at SLEEP in 2011. They found that independently of OSA the treatment of insomnia decreased the sleep latency by 36 min and increased the total sleep time by 0.7 h and the sleep efficiency by 18% (36).

Given the sparse evidence and the inconsistency of the current evidence, more research is needed in this topic. Nonetheless, the general conclusion from the study by Sweetman et al. is that both groups, namely patients with insomnia only and COMISA, benefited in their sleep and daytime measures from CBTi about equally well (21). Thus having co-morbid OSA did not impair the treatment of the insomnia in the COMISA group.

Several studies investigated whether the presences of comorbid insomnia impaired PAP-adherence but the inconsistent results make it difficult to conclude that comorbid insomnia impairs PAP adherence. To answer this question a randomized controlled trial should be performed in the future with a group of patients treated with PAP alone and another group treated with a combination of PAP and CBTI, e.g., in the form suggested by Crawford et al. (37).

There is evidence that insomnia can be treated also in COMISA patients with CBTI (21). That, in itself may reduce the disease burden and justify the use of CBTI, even if it does not improve PAP adherence in the OSA treatment. However, two studies are few and more studies are needed. Especially studies pre-treating or concurrently treating the insomnia in COMISA patients deserve to be thoroughly assessed. Even more, the findings of our review are inconsistent with those of Luyster et al., who found one study with a remarkable effect of CBTI in OSA patients, namely an increased PAP-adherence with CBTI in OSA with an OR of 6 (35).

As a few studies suggest that OSA-treatment has an impact on insomnia, an immediate PAP-therapy and reevaluation of insomnia symptoms in the course of it, might lead to insomnia improvement or abandonment of CBTI due to subclinical severity scores.

The revision of studies on PAP-adherence revealed several attempts to build adherence groups, which is an indicator of missing thresholds for PAP-adherence evaluation. We recommend a definition of adherence to PAP-therapy in hours per night or nights of use per months, since it would increase the comparability of the results and might ease the decision whether or not an intervention is reasonable.

The time pattern of insomnia symptoms, namely middle insomnia with awakenings during the nights, initial insomnia with difficulties to fall asleep and late insomnia, which is to say early morning awakenings, seem to be entirely different COMISA entities that react differently to PAP-therapy and might have different effects on PAP-therapy-adherence. Such a phenotypic classification may prove to be quite useful in future studies in order to provide personalized care in this broad group of patients.

Reviewing the literature, it has been interesting to see, that pneumologists and sleep physicians put emphasis on apneic events, whereas neurologists and psychologists focused more on insomnia criteria. Therefore, we suggest an interdisciplinary approach in the treatment of COMISA patients in order to reflect on both entities equally.

Due to the above mentioned inconsistency of study designs and results the temporary evidence level is not yet sufficient for a meta-analysis on COMISA therapy. In order to promote personalized management to COMISA patients and to perform meta-analyses in the future, it might be advisable to use study designs that specify both insomnia in the time domain (initial, middle, late) and that consider CBTI as well as PAP-therapy regimens in distinct combined patient phenotypes.

## Author contributions

IT reviewed the part of COMISA and CBTI as well as Table 2. RC is responsible for the study design, provided data extraction from the original literature, analysis, presentation, and interpretation of data eligibility criteria and draft the flow chart. HG is responsible for study conception and design, helped on the introduction and the discussion. KB reviewed the part of COMISA and PAP-therapy, created tables, created introduction, conclusion, and parts of the discussion. All authors approved the submitted version of the manuscript and take full responsibility for it.

### Conflict of interest statement

The authors declare that the research was conducted in the absence of any commercial or financial relationships that could be construed as a potential conflict of interest.
