# Supplementary material for: Current Treatment of Comorbid Insomnia and Obstructive Sleep Apnea With CBTI and PAP-Therapy: A Systematic Review
Source: Front Neurol. 2018 Oct 29;9:804. doi: 10.3389/fneur.2018.00804 (PMC6215826; doi:10.3389/fneur.2018.00804)
Supplement: Supplementary file 1 [file Data_Sheet_1.pdf]

MOOSE Checklist

From: Stroup, D. et al. (2000). Meta-analysis of observational studies in epidemiology. A proposal for reporting. Journal of the American Medical Association, 283(15):2008-2012.doi: 10.1001/jama.283.15.2008

	Reported on
**Reporting of background should include**	
Problem definition	Introduction, paragraphs 1 to 3
Hypothesis statement	Introduction, paragraphs 4 & 5
Description of study outcomes	Methods, paragraph 1
Type of exposure or intervention used	Methods, paragraph 1
Type of study designs used	Methods, paragraph 1
Study population	Methods, paragraph 1
**Reporting of search strategy should include**	
Qualifications of searchers (eg librarians/investigators)	Methods, paragraph 2
Search strategy, including time period used in the synthesis and key words	Methods, paragraph 2
Effort to include all available studies, including contact with authors	Discussion, paragraph 4
Databases and registries searched	Methods, paragraph 2; discussion, paragraph 4
Search software used, name and version, including special features used (eg explosion)	Methods, paragraph 2
Use of hand searching (eg reference lists of obtained articles)	Methods, paragraph 2; Results, paragraph 1 (flow diagram)
List of citations located and those excluded, including justification	Results, paragraph 1
Method of addressing articles published in languages other than English	Methods, paragraph 2; results, paragraph 1 (flow diagram)
Method of handling abstracts and unpublished studies	Discussion, paragraph 4
Description of any contact with authors	Discussion, paragraph 4
**Reporting of methods should include**	
Description of relevance or appropriateness of studies assembled for assessing the hypothesis to be tested	Results, paragraphs 1 & 2 (tables 1 and 2); discussion, paragraphs 1 to 3
Rationale for the selection and coding of data (eg sound clinical principles or convenience)	Methods, paragraph 3; results, paragraphs 1 & 2 (tables 1 and 2); discussion, paragraph 4
Documentation of how data were classified and coded	Methods, paragraph 3; discussion, paragraph 4

(eg multiple raters, blinding and interrater reliability)	
Assessment of confounding (eg comparability of cases and controls in studies where appropriate)	Results, paragraphs 1 to 3 (tables 1 and 2); discussion, paragraphs 1 to 3
Assessment of study quality, including blinding of quality assessors, stratification or regression on possible predictors of study results	Results, paragraphs 1 to 3 (tables 1 and 2); discussion, paragraphs 1 to 3
Assessment of heterogeneity	Results, paragraphs 1 to 3 (tables 1 and 2); discussion, paragraphs 1 to 3
Description of statistical methods (eg complete description of fixed or random effects models, justification of whether the chosen models account for predictors of study results, dose-response models, or cumulative meta-analysis) in sufficient detail to be replicated	Not applicable.
Provision of appropriate tables and graphics	Results, paragraphs 1 & 2 (figure 1, tables 1 and 2)
**Reporting of results should include**	
Graphic summarizing individual study estimates and overall estimate	Not applicable.
Table giving descriptive information for each study included	Results, paragraphs 1 & 2 (tables 1 and 2)
Results of sensitivity testing (eg subgroup analysis)	Not applicable.
Indication of statistical uncertainty of findings	Results, paragraphs 1 & 2 (tables 1 and 2)
**Reporting of discussion should include**	
Quantitative assessment of bias (eg publication bias)	Not applicable.
Justification for exclusion (eg exclusion of non-English language citations)	Results, paragraph 1 (flow diagram)
Assessment of quality of included studies	Results, paragraphs 1 to 3 (tables 1 and 2); discussion, paragraphs 1 to 3
**Reporting of conclusions should include**	
Consideration of alternative explanations for observed results	Discussion, paragraphs 1 to 3
Generalization of the conclusions (eg appropriate for the data presented and within the domain of the literature review)	Discussion, paragraphs 8 to 14
Guidelines for future research	Discussion, paragraphs 8 to 14
Disclosure of funding source	There is none (see conflict of interest statement)
